# Supplementary material for: Clinical Post-SARS-CoV-2 Infection Scenarios in Vaccinated and Non-Vaccinated Cancer Patients in Three German Cancer Centers: A Retrospective Analysis
Source: Cancers (Basel). 2022 Jul 31;14(15):3746. doi: 10.3390/cancers14153746 (PMC9367483; doi:10.3390/cancers14153746)
Supplement: Supplementary file 1 [file cancers-14-03746-s001.zip › cancers-1802976-supplementary.pdf]

Supplemental Table S1: Vaccination status of the 133 cancer patients of this study before acquiring a SARS-CoV-2 infection.

**COVID-19 vaccination status**		
**Vaccinated patients (*n* and %)**	49/133	37%
**Data of the vaccinated patients**	*n* = 49	100%
**Type of first vaccination**		
Vaxzevria AZD1222	4	8%
Comirnaty BNT162b2	36	72%
Spikevax mRNA-1273	2	4%
Johnson and Johnson Ad26.COV2.S	2	4%
Missing	5	5%
**Type of second vaccination**	48/49	
Vaxzevria AZD1222	1	2%
Comirnaty BNT162b2 or Spikevax mRNA-1273	3	6%
Comirnaty BNT162b2	38	79%
Missing	6	13%
**Summary of COVID-19 vaccines among double vaccinated patients**	48/49	98%
Vector based vaccine followed by mRNA	4	8%
Vector based only	1	2%
mRNA based only	37	77%
Missing	6	13%
Third vaccination (mRNA based only)	17/49	35%
Median time from vaccination until SARS-CoV-19 infection in days (range)	129	2-268
